# Unnoticed metallic foreign body in the camerular angle inducing chronic uveitis

**DOI:** 10.5935/0004-2749.20210038

**Published:** 2021

**Authors:** Juliana Albano, Maria Campos Pires, Marcelo Paccola

**Affiliations:** 1 Department of Ophthalmology, Faculty of Medical Sciences, Universidade Estadual de Campinas, Campinas, SP, Brazil; 2 Faculty of Medicine, Universidade de Itaúna, Itaúna, MG, Brazil

**Keywords:** Eye foreign bodies, Uveitis, anterior, Uveitis, intermediate, Corneal edema, Toxocariasis, Corpos estranhos no olho, Uveíte anterior, Uveíte intermediária, Edema da córnea, Toxocaríase

## Abstract

We report the case of an eight-year-old male patient with a four-month history of unilateral anterior chronic uveitis, associated with a pigmented lesion surrounded by fibrinoid material in the inferior camerular angle and with a fibrotic lesion in the extreme periphery of the inferior retina. The patient had no history of trauma or any other clinical symptoms. Although the patient was suspected of having toxocariasis, serological tests were negative. Partial symptomatic improvement was achieved using both orally and topically administered corticosteroids. In addition, a decrease in fibrinoid material around the pigmented camerular lesion revealed it to be regular and cylindric. Computed tomography of the orbits revealed a metallic foreign body in the topography of the inferior camerular angle. The patient underwent removal of the foreign body through a corneal incision and photocoagulation around the inferior retinal traction. Excellent visual and anatomical results were obtained.

## Abstract (pt)

Os autores relatam o caso de paciente do sexo masculino, 8 anos de idade, com história de uveíte crônica anterior unilateral há quatro meses, associada a lesão pigmentada envolvida por material fibrinóide em ângulo camerular inferior e a lesão fibrótica em extrema periferia de retina inferior. Não havia histórico de trauma ou outros sintomas clínicos. A hipótese de toxocaríase foi afastada diante de testes sorológicos negativos. Melhoria sintomática parcial foi alcançada com administração de corticosteróide vias oral e tópica. Ademais, redução na quantidade de material fibrinóide ao redor da lesão camerular a revelou regular e cilíndrica. Foi realizada tomografia computadorizada de órbitas, permitindo a detecção de corpo estranho metálico na topografia de ângulo camerular inferior. O paciente foi submetido a remoção do corpo estranho através de incisão cor neana e a fotocoagulação ao redor da tração retiniana inferior. Excelentes resultados visual e anatômico foram obtidos.

## INTRODUCTION

Intraocular foreign body (IOFB) is a relatively frequent finding associated with penetrating trauma. However, in the absence of trauma history, this diagnosis is uncommon and challenging. Inert materials that cause little inflammatory response may take a variable amount of time to be discovered, and treatment depends on the consequences of their presence in the eye. Treatment may be expectant in the absence of inflammation, retinal traction, detachment, elevated intraocular pressure, chronic corneal edema, or toxic neuropathy, or it may be surgical in the presence of one of these complications^(1-3)^.

We report a case of an IOFB located at the lower angle of the anterior chamber in an eight-year-old boy with no history of trauma that evolved with anterior chamber inflammation and corneal decompensation and was managed surgically with excellent visual and anatomical results.

## CASE REPORT

**Figure 1:**
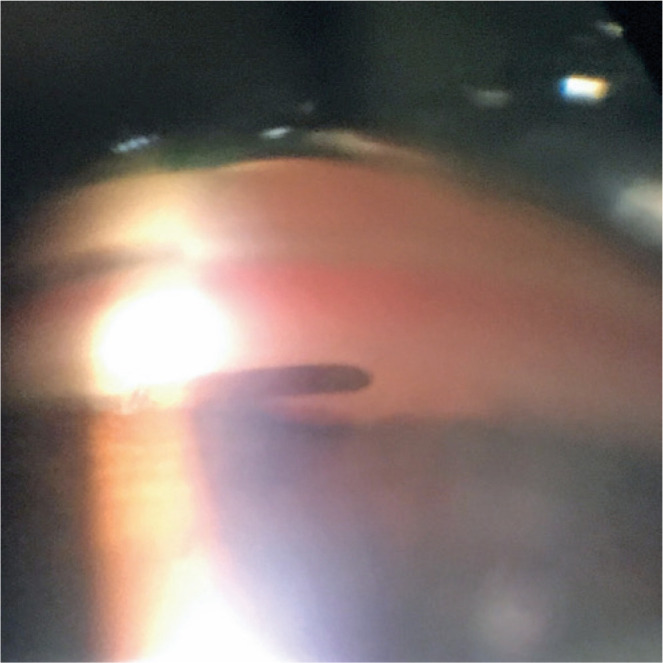
Inferior camerular angle showing the pigmented lesion without associated anterior synechiae.

An eight-year-old boy was brought by his mother to the clinic with complaints of red eye, photophobia, and pain in the right eye for about four months, with sporadic improvements associated with the use of eyedrops of ciprofloxacin with dexamethasone. The patient had no history of trauma, comorbidities, or allergies, and other ocular and systemic symptoms were negative. On examination, the patient’s visual acuity was 20/30 in the right eye and 20/15 in the left eye. Biomicroscopy demonstrated mild conjunctival hyperemia, epithelial corneal edema, and anterior chamber reaction of 2+/4+. A pigmented lesion was noticed in the inferior camerular angle, which was associated with surrounding fibrinoid material. No adjacent anterior synechiae were seen on gonioscopy (Figure 1), whereas on fundoscopy, a lesion in the extreme inferior periphery of the retina was detected through indentation, in association with local retinal traction, without retinal detachment. Ocular ul trasound did not reveal any alteration.

**Figure 2:**
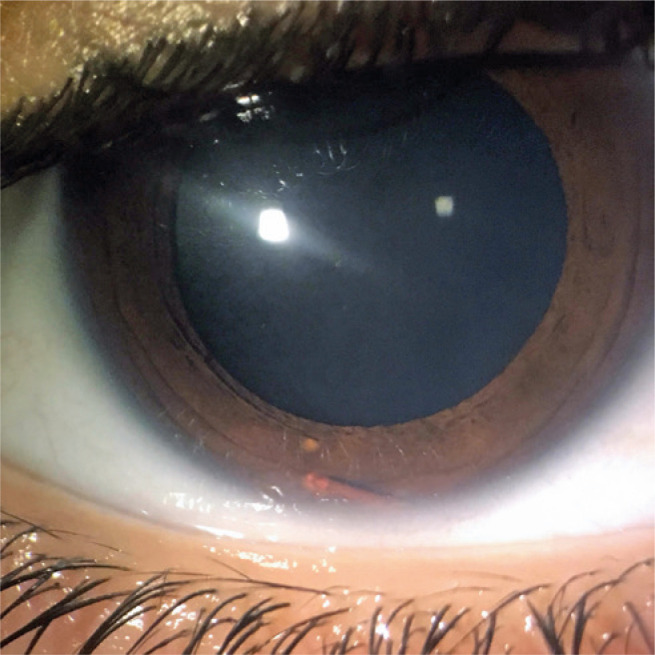
Anterior segment biomicroscopy showing the cylindric and regular pigmented lesion in the inferior part of the anterior chamber.

Toxocariasis with secondary peripheral retinal granuloma was hypothesized, and topical treatment with tropicamide and prednisolone was initiated in addition to oral antiparasitic treatment. Laboratory tests, including intradermal tuberculosis test; serological tests for toxoplasmosis, toxocariasis, and syphilis; and blood cell counts, resulted in negative values or values within the reference range. After two weeks with no improvement, prednisone was introduced orally with maintenance of topical medications. After two additional weeks, the surrounding fibrinoid material was absorbed, revealing a regular cylinder-shaped camerular lesion (Figure 2). Faced with a diagnostic hypothesis of a metallic IOFB, orbit radiography was performed.

**Figure 3:**
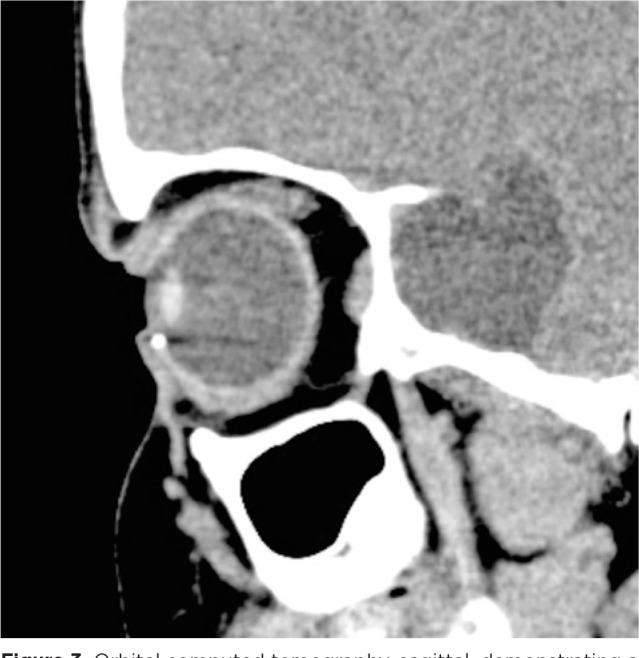
Orbital computed tomography, sagittal, demonstrating a metallic foreign body in a topography compatible with the inferior camerular angle of the right eye.

**Figure 4:**
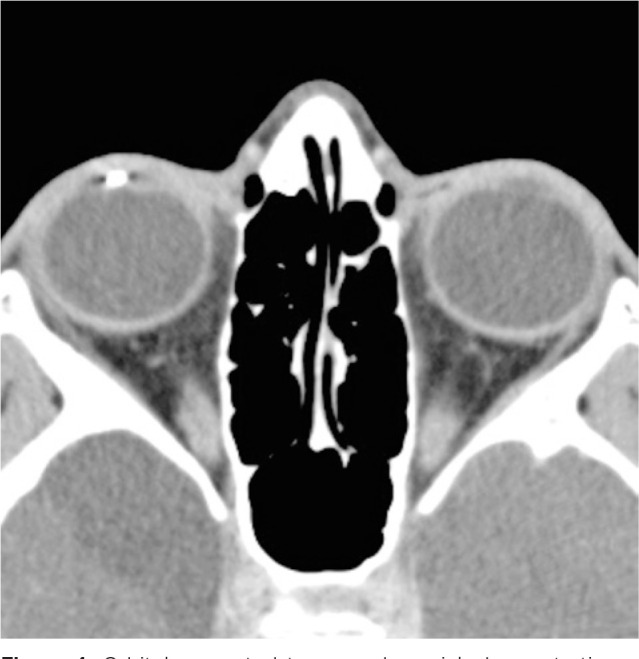
Orbital computed tomography, axial, demonstrating a metallic foreign body in a topography compatible with the inferior camerular angle of the right eye.

Radiography showed an image suggestive of IOFB, but details could not be assessed. Therefore, computed tomography of the orbits was performed, which provided evidence of a metallic and cylindric IOFB in the anterior chamber topography (Figures 3 and 4).

The patient was referred for surgery. A 2-mm metallic fragment was removed through a corneal incision 180° from the IOFB location, with no complications. Taking into consideration the oblique position of the IOFB at the angle, it was hypothesized that its trajectory would have been from the superotemporal and anterior to inferonasal and posterior, reaching the eye in the inferior corneal limbus.

On the 15^th^ postoperative day, anterior segment changes, such as corneal edema, ocular hyperemia, cham ber reaction, and corectopy, were no longer observed. However, a horseshoe-shaped retinal tear was observed through indented fundoscopy at the extreme inferior periphery of the retina, adjacent to the previously seen vitreoretinal traction. Perilesional photocoagulation was performed around the tear while avoiding tractional areas. The patient’s final visual acuity was 20/15 bilaterally, and no new symptoms or changes emerged.

## DISCUSSION

IOFB is most often diagnosed in the immediate posttrauma period and is treated with surgical removal. However, the IOFB may go unnoticed, and the story may not be enlightening regarding the occurrence of penetrating trauma. In such cases, complications may arise including infection, corneal decompensation, recurrent uveitis, glaucoma, iron siderosis, copper chalcosis, and secondary optic atrophy, cases in which early diagnosis and management are essential for a better prognosis^(1,2)^.

Signs indicating the long-term presence of an IOFB in the anterior chamber involve pigmented keratic precipitates in the endothelium, anterior chamber reaction, corneal edema, or IOFB pigmentation itself, corresponding to the case in question^(1)^. Inert materials such as plastic, glass, gold, silver, and platinum may be inert for a long time before presentation because of minimal excitation of the inflammatory response^(1,3-5)^. In these cases, the history of the trauma may be old and sometimes not remembered by the patient, adding difficulty to the diagnosis. Mahmoud et al. described a case of a woody IOFB in the anterior chamber after vegetable trauma one year earlier, with recurrent episodes of hyperemia and ocular pain, which partially improved after topical corticosteroid therapy. The granuloma around the wood fragment resolved with corticotherapy, which was followed by surgical removal of the IOFB^(6)^.

The angle location of the anterior chamber may especially be hidden because of the presence of corneal edema, pterygium, or peripheral corneal vascularization. Gonioscopy and ultrasound biomicroscopy are important diagnostic tools for proper localization of the IOFB. In addition, anterior segment optical coherence tomography, orbital radiography, or orbital computed tomography are helpful, especially in cases of metallic IOFB^(1,7)^.

When complications are present, surgical removal of the IOFB becomes imperative. The approach depends on the location of the IOFB and could be via the anterior segment, with or without the aid of specific lenses for proper visualization, or the posterior segment, through vitrectomy and forceps or a magnetic instrument, in the case of metallic materials. Management of complications should not be overlooked, including photocoagulation in cases of retinal tears, management of intraocular pressure to prevent glaucoma and phacoaspiration in cases of crystalline involvement, and development of cataract^(1,2)^.

Patient prognosis generally depends on the location of the IOFB and the resulting complications, which are worse in cases that involve the macula, optic nerve or central cornea, and in cases with associated complications such as retinal detachment, significant elevation of intraocular pressure, or corneal decompensation. Early and proper management contribute to a better vision prognosis^(1,2)^.

There should be a high index of suspicion regarding the presence of IOFBs in the context of chronic unilateral anterior uveitis. This is especially true for children, for which a related history of trauma is often absent. Adequate diagnosis and management are of paramount importance to provide good final visual and anatomical results.
